# Nitric Oxide-Releasing Drug Glyceryl Trinitrate Targets JAK2/STAT3 Signaling, Migration and Invasion of Triple-Negative Breast Cancer Cells

**DOI:** 10.3390/ijms22168449

**Published:** 2021-08-06

**Authors:** Sarra Bouaouiche, Silvia Ghione, Randa Sghaier, Olivier Burgy, Cindy Racoeur, Valentin Derangère, Ali Bettaieb, Stéphanie Plenchette

**Affiliations:** 1 Laboratoire d’Immunologie et Immunothérapie des Cancers (LIIC), EPHE, PSL Research University, 75000 Paris, France; bouaouiche.sarra@gmail.com (S.B.); silvi.ghio50@gmail.com (S.G.); sg.randa@yahoo.fr (R.S.); Cindy.Racoeur@u-bourgogne.fr (C.R.); ali.bettaieb@u-bourgogne.fr (A.B.); 2 Laboratoire d’Immunologie et Immunothérapie des Cancers (LIIC), EA7269, Université de Bourgogne Franche-Comté, 21000 Dijon, France; 3 INSERM U1231, UFR Sciences de Santé, Université de Bourgogne-Franche Comté, 21000 Dijon, France; Olivier.Burgy@u-bourgogne.fr; 4 Plateforme de Transfert en Biologie du Cancer, Centre Georges-François Leclerc, 21000 Dijon, France; VDerangere@cgfl.fr

**Keywords:** cancer, nitric oxide, signaling, migration, invasion, metastasis

## Abstract

Triple-negative breast cancer (TNBC) is a highly aggressive disease with invasive and metastasizing properties associated with a poor prognosis. The STAT3 signaling pathway has shown a pivotal role in cancer cell migration, invasion, metastasis and drug resistance of TNBC cells. IL-6 is a main upstream activator of the JAK2/STAT3 pathway. In the present study we examined the impact of the NO-donor glyceryl trinitrate (GTN) on the activation of the JAK2/STAT3 signaling pathway and subsequent migration, invasion and metastasis ability of TNBC cells through in vitro and in vivo experiments. We used a subtoxic dose of carboplatin and/or recombinant IL-6 to activate the JAK2/STAT3 signaling pathway and its functional outcomes. We found an inhibitory effect of GTN on the activation of the JAK2/STAT3 signaling, migration and invasion of TNBC cells. We discovered that GTN inhibits the activation of JAK2, the upstream activator of STAT3, and mediates the S-nitrosylation of JAK2. Finally, the effect of GTN (Nitronal) on lung metastasis was investigated to assess its antitumor activity in vivo.

## 1. Introduction

Triple-negative breast cancer (TNBC) is the most aggressive subtype of breast cancer among women worldwide. TNBCs represent 10–15% of all subtypes of breast cancers and are so named because of the absence of estrogen receptor (ER), progesterone receptor (PR) and no overexpression of human epidermal growth factor receptor 2 (HER2) [1]. Consequently, standard targeted therapies used for treating other subtypes of breast cancers (hormone receptor-positive or HER2-positive) are not a therapeutic option. The therapeutic care of patients afflicted with early or advanced TNBC remains chemotherapy. Although TNBC patients present higher chemosensitivity than for other subtypes of breast cancer, their prognosis remains worse [2]. In recent years, much emphasis has been put on TNBC molecular features to identify new therapeutic targets and options of treatment.

The signal transducers and activators of transcription (STAT) family encompasses key transcription factors involved in breast cancer disease. Importantly, STAT3 signaling pathways, activated through many cytokines (such as Interleukin-6 (IL-6), IL-10) and growth factors (epidermal growth factor (EGF), fibroblast growth factor (FGF), insulin-like growth factor (IGF)), promote breast cancer progression, proliferation, apoptosis, metastasis and chemoresistance [3]. It is well established that the tumor microenvironment plays a crucial role for tumor development, tumor progression and response to anticancer therapies [4,5]. A continuous dynamic cross talk between tumor cells and stroma cells accounts tremendously for tumor fate and patient prognosis. A variety of stroma cells including immune cells (from the innate and adoptive response), cancer-associated fibroblasts (CAFs) and blood vasculature mainly populate the tumor microenvironment [4,6]. Inflammatory cytokines, chemokines and growth factors are active players in the communications within the tumor microenvironment that support tumor growth, invasion, migration and initiate the metastatic cascade [7].

IL-6 is a major pro-inflammatory cytokine secreted in the tumor microenvironment from both tumor and stromal cells which influences almost all hallmarks of cancer to promote cancer growth and progression [8]. The autocrine and paracrine action of IL-6 is associated with multiple signaling pathways that support not only aggressive features of cancer cells such as invasion, migration and metastasis, but also tumor growth, survival angiogenesis, regulation of immune response and chemoresistance [9]. Tumor-associated macrophages (TAMs), cancer-associated fibroblasts (CAFs) or myeloid-derived suppressor cells (MDSCs) are major sources of IL-6 and play an important role in invasiveness and metastasis [10,11]. Various clinical studies have correlated high circulating IL-6 levels in patients with malignant tumors, as for breast cancer [12,13], prostate cancer [14], head and neck squamous cell carcinoma [15] or renal cancer [16]. Even though several studies positively correlated elevated serum IL-6 levels with a poor prognosis, further studies are required to clearly determine whether IL-6 is a cause or consequence in different types of cancer [17]. Nevertheless, in breast cancer, elevated systemic IL-6 seems to reflect poor prognosis, advanced disease and distant metastasis [13]. The classical and alternative IL-6 signaling pathways activate JAKs with subsequent activation of the signal transducer and activator of transcription-3 (STAT3), a key transcription factor inducing numerous effector genes involved in cancer promotion and malignancy [18,19,20]. IL-6/STAT3 activity is also often associated with metastatic processes including epithelial-mesenchymal transition (EMT), degradation of extracellular matrix and cell migration [20]. Typically, IL-6 exerts its effect through two functional receptor complexes composed of a transmembrane form IL-6 receptor alpha (mIL-6 Rα) or a soluble form (sIL-6 R) that provides IL-6 binding specificity and interaction with the signal-transducing chains (gp130). JAK1 and JAK2 are critical tyrosine kinases that trigger the phosphorylation and activation of STAT3 on tyrosine 705 (Tyr 705) and subsequent dimer formation that initiates its translocation into the nucleus [19]. The IL-6/JAK2/STAT3 axis represents an important therapeutic target [18]. IL-6 also mediates its effect through the activation of other oncogenic signaling pathways including PI3K/AKT and MAPK/ERK pathways [8]. Thus, neutralizing either IL-6 or related signaling pathways represent an attractive therapeutic target in cancer. However, anti-IL-6 therapies have mainly demonstrated no benefit in several types of cancer [21]. Hence, new anticancer strategies targeting the IL-6 signaling axis are needed. Here, we considered a nitric oxide (NO)-based strategy to prevent the IL-6/JAK2/STAT3 axis. NO is an important signaling molecule now understood to play a dual role in cancer as a positive or negative regulator of multiple signaling pathways [22]. Some phase II clinical trials in advanced non-small cell lung cancer and prostate cancer patients reveal that the use of glyceryl trinitrate (GTN) may have beneficial effects in combination with chemotherapy (vinorelbine and cisplatin) and/or radiotherapy [23,24]. Interestingly, cisplatin can increase IL-6 cytokine production and then cellular migration and proliferation in non-small cell lung cancer cells [25]. However, further phase III trials in advanced non-small cell lung cancer patients treated with platinum-based doublet as first-line therapy (carboplatin/gemcitabine or carboplatin/paclitaxel) with GTN did not present clinical benefit [26]. Better knowledge is required to define how GTN should be utilized in cancer therapy.

In the present study we investigated the effect of carboplatin in promoting IL-6-mediated TNBC cell migration. We then examined the ability of GTN, a NO-releasing drug, to counteract the IL-6/JAK2/STAT3 axis migration, invasion and metastasis of TNBC cells.

## 2. Results

### 2.1. Effect of Carboplatin on the Production of IL-6 in TNBC Cells

**Figure 1:**
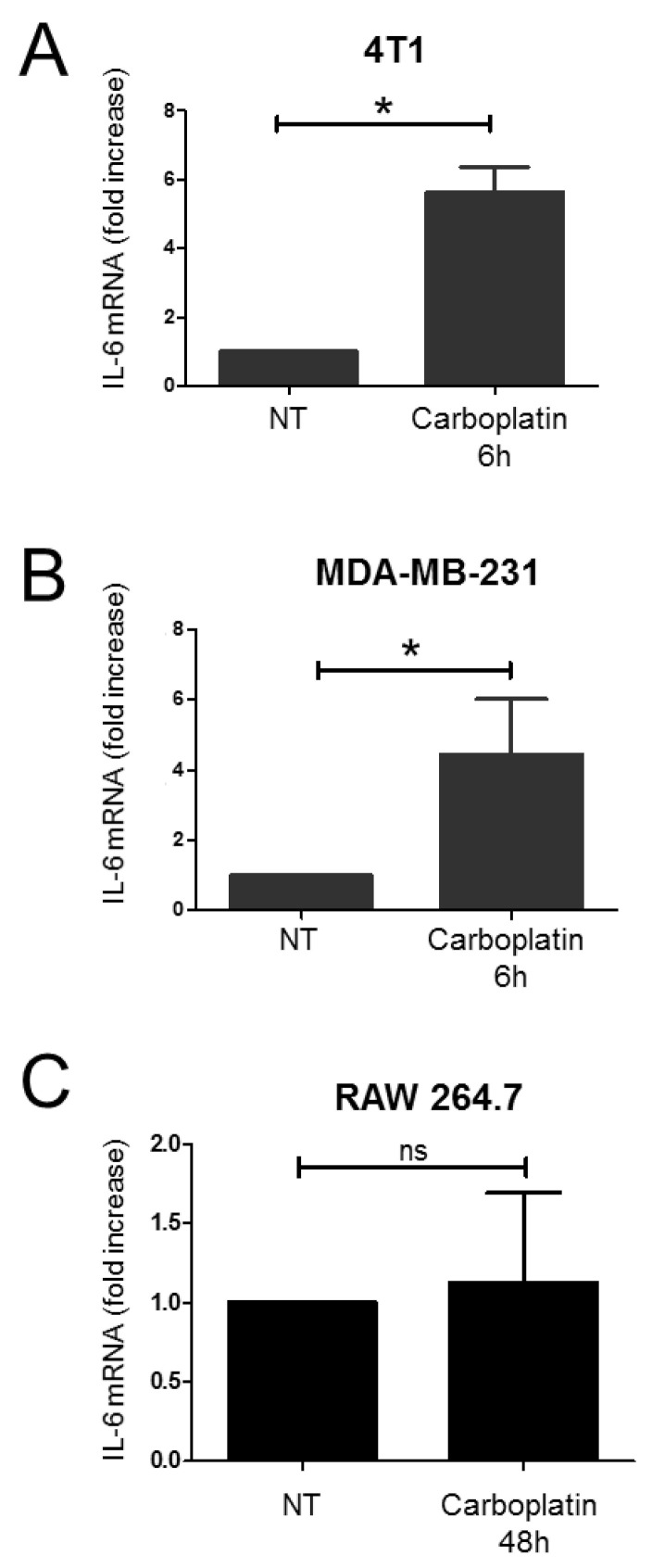
Carboplatin increases IL-6 production in TNBC cells. (**A**) 4T1 cells, (**B**) MDA-MB-231 cells and (**C**) RAW 264.7 cells were treated with carboplatin (10 µM, 750 nM and 10 µM, respectively) at the indicated time and IL-6 mRNA levels were determined by RT-qPCR. Data are shown as fold increase with mean +/− SD of three independent experiments performed in duplicate. *p* values were determined using a one-tailed Mann–Whitney test. Statistically significant differences are indicated: * *p <* 0.05; ns: not significant.

Firstly, we investigated the effect of carboplatin on the expression of the pro-inflammatory cytokine IL-6 in TNBC cells from mouse (4T1 cells) and human (MDA-MB-231) origin. The 4T1 cells and MDA-MB-231 cells were exposed to a subtoxic concentration of carboplatin for 6 h (Supplementary Figure S1). We found that carboplatin induces an increase in IL-6 at the mRNA level in TNBC cells. A significant augmentation of the level of IL-6 mRNA was found in 4T1 cells after 6 h of treatment with carboplatin (Figure 1A). Similarly, we found that carboplatin induces the expression of IL-6 in MDA-MB-231 cells after 6 h of treatment (Figure 1B). Exposure of RAW 264.7 cells to a subtoxic concentration of carboplatin showed no significant changes in IL-6 mRNA levels, neither after 48 h of treatment (Figure 1C), nor at 6 h and 24 h (data not shown).

### 2.2. GTN Inhibits JAK2/STAT3 Signaling Pathway

**Figure 2:**
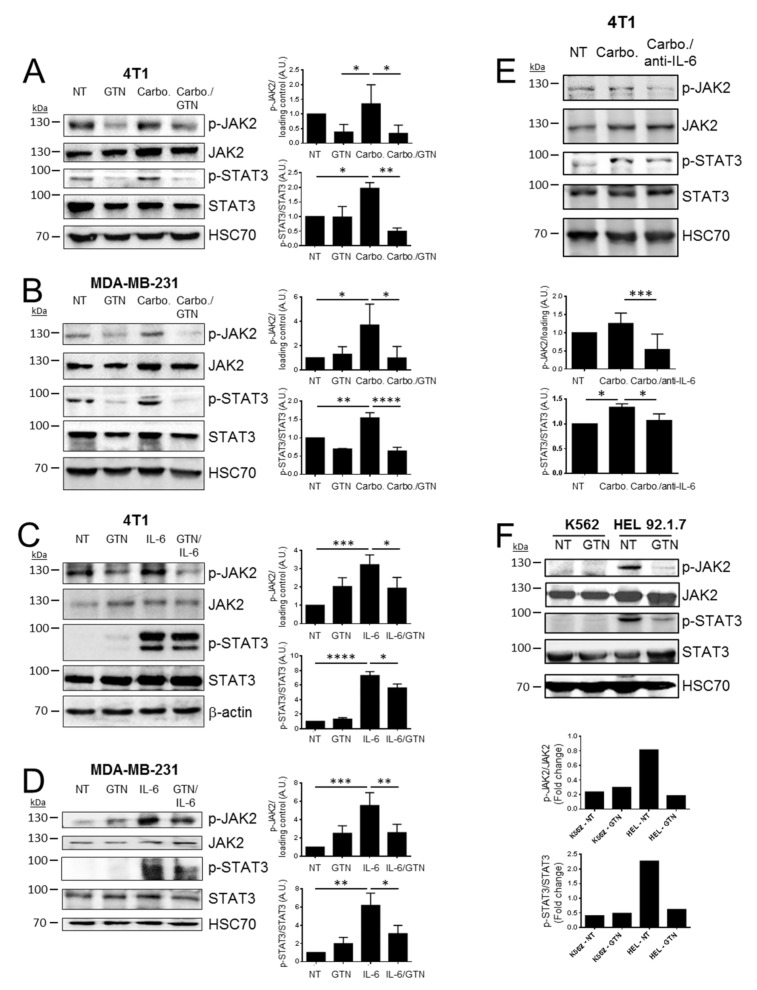
GTN inhibits IL-6-mediated JAK2/STAT3 signaling in TNBC cells. (**A**) 4 T1 cells and (**B**) MDA-MB-231 cells were treated with glyceryl trinitrate (GTN, 250 µM) and/or carboplatin (10 µM for 4T1 cells, 750 nM for MDA-MB-231 cells) or left untreated (non-treated, NT) for 48 h. (**C**) 4T1 cells and (**D**) MDA-MB-231 cells were treated with GTN (250 µM), recombinant murine or human IL-6 (rec-IL-6, 10 ng/mL), rec-IL-6/GTN or left untreated (non-treated, NT) for 6 h and 24 h respectively. (**E**) 4T1 cells were treated with carboplatin in presence or absence of neutralizing IL-6 antibody (anti-IL-6), or left untreated (non-treated, NT). (**F**) K562 and HEL 92.1.7 were treated with GTN 250 µM for 48 h. (**A**–**F**) The levels of phosphorylated JAK2 (p-JAK2), total JAK2, phosphorylated STAT3 (p-STAT3) and total STAT3 were analyzed by Western blot. HSC70 or β-actin was used as a loading control in all Western blots performed. The blots presented are representative of at least three independent experiments. (**A**–**E**) The histograms present quantification using densitometric analyses of at least three independent experiments: mean ratio p-JAK2/loading control (p-JAK2 normalized to the loading control either HSC70 or β-actin due to non-specific increase in JAK2 basal levels in 4T1 cells in response to treatment) and mean ratio p-STAT3/STAT3. (**F**) The histograms present quantification of one representative experiment (from three independent experiments). Statistically significant differences were determined using a one-way ANOVA test (with a Fisher’s LSD test). * *p <* 0.05; ** *p <* 0.01; *** *p* < 0.0001; **** *p* < 0.00001. (**E**) Statistical analysis for ratio p-STAT3/STAT3: * *p* < 0.1.

The JAK2/STAT3 signaling pathway can be activated by IL-6. By immunoblotting analyses, we confirmed that the JAK2/STAT3 signaling pathway, in 4T1 and MDA-MB-231 cells, is activated as attested by the phosphorylation of JAK2 (Tyr1007/1008) and STAT3 (Tyr705), either by carboplatin or recombinant IL-6 treatment (Figure 2A–D). We investigated the effect of the NO donor GTN on the JAK2/STAT3 signaling pathway activation following carboplatin or recombinant IL-6 treatment. GTN induces the release of NO as attested by the increase in nitrite (NO_2_^−^) levels. This effect is not modified upon carboplatin exposure (Supplementary Figure S2). GTN induces a modest cytotoxic effect against 4T1 and MDA-MB-231 cells after 48 h, but not after 24 h exposure (Supplementary Figure S3). In agreement, GTN has no significant impact on early apoptosis in TNBC cells (4T1 and MDA-MB-231) after 24 h and 48 h of treatment. It induces only a low amount of early/late apoptosis in MDA-MB-231 cells after 48 h treatment (Supplementary Figure S4). GTN treatment in combination with either carboplatin or IL-6 significantly decreased p-JAK2 and subsequent p-STAT3 in TNBC cells (Figure 2A–D). To confirm the capacity of carboplatin to activate the JAK2/STAT3 signaling pathway through IL-6, we treated 4T1 cells with carboplatin with or without an IL-6 neutralizing antibody. Immunoblot analysis revealed that elevated levels of p-STAT3 and p-JAK2 stimulated by carboplatin can be blocked by the IL-6 neutralizing antibody and confirm the role of carboplatin in mediating IL-6 production and JAK2/STAT3 activation (Figure 2E).

These results suggest that GTN can inhibit the activation of the JAK2/STAT3 signaling pathway. We further explored the effect of NO on the phosphorylation state of JAK2. We therefore employed the erythroleukemic cell line HEL 92.1.7, characterized by the single point mutation JAK2-V617F, a gain-of-function mutant form of JAK2, versus the erythroleukemic cell line K562 that contains the non-mutated form of JAK2. As expected, a high level of basal expression of p-JAK2 was observed in the HEL 92.1.7 cell line as compared to K562 cells. Treatment of HEL 92.1.7 cells with GTN results in a significant decrease in p-JAK2 and subsequent diminution of p-STAT3 (Figure 2F).

Taken together, these results indicated that NO can counteract the JAK2/STAT3 pathway via the inhibition of p-JAK2.

### 2.3. NO Promotes JAK2 S-Nitrosylation

**Figure 3:**
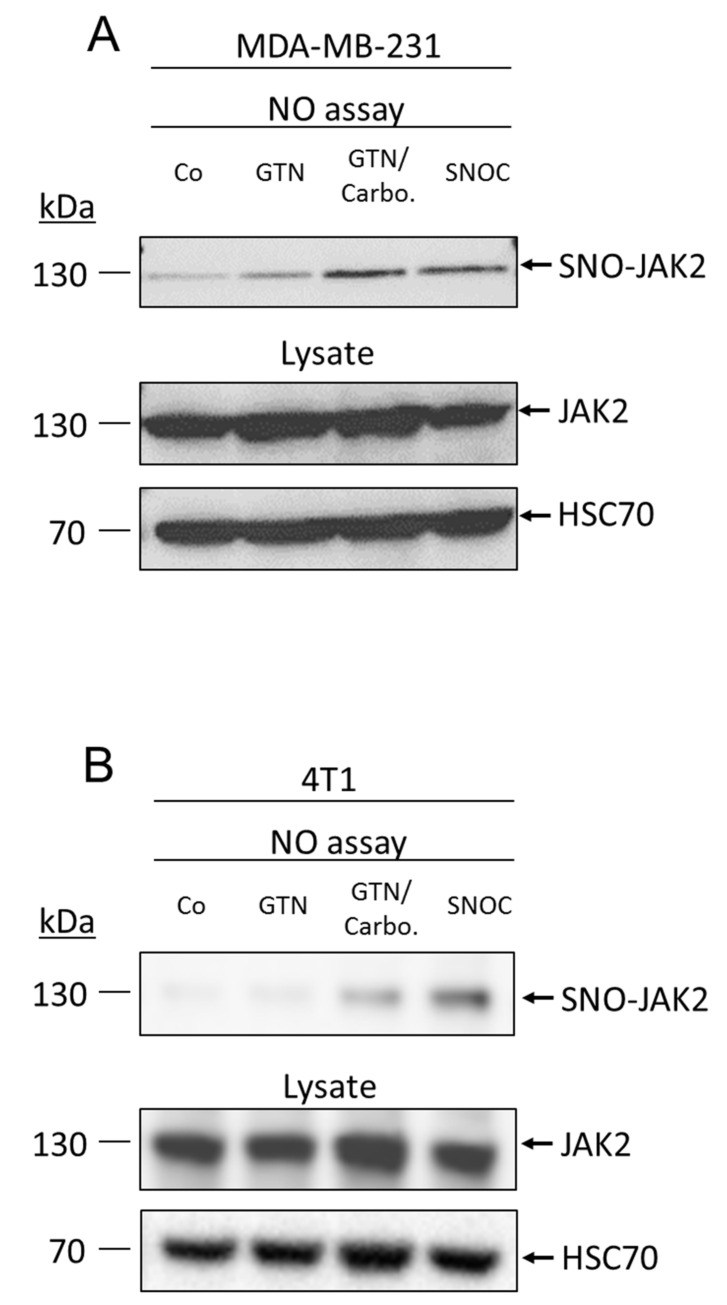
GTN promotes JAK2 S-nitrosylation. (**A**) MDA-MB-231 cells or (**B**) 4T1 cells were treated with carboplatin (750 nM and 10 µM, respectively) and/or GTN (250 µM) for 24 h, and S-nitrosocysteine (SNOC 1 mM) for 15 min. S-nitrosylation of JAK2 was demonstrated by using the biotin switch assay method. Total lysates were immunoblotted with JAK2 and HSC70 as a loading control.

A myriad of signaling pathways regulated by NO occur through protein post-translational modification by S-nitrosylation. We investigated whether NO donors could promote the S-nitrosylation of JAK2 that may inhibit its kinase activity and downstream activation of the signaling pathway. To examine whether JAK2 can be S-nitrosylated, MDA-MB-231 and 4T1 cells were treated with GTN, with or without carboplatin, or another NO donor (S-nitrosocysteine (SNOC)) and then subjected to biotin-switch assay. We identified JAK2 as a target of NO. Interestingly, GTN alone only slightly induces the S-nitrosylation of JAK2, as seen only in MDA-MB-231 cells (Figure 3A). Interestingly, in both MDA-MB-231 and 4T1 cell lines, the combination therapy GTN/carboplatin markedly induced the S-nitrosylation of JAK2 (Figure 3A,B). In addition, SNOC (massive and rapid delivery of NO) induced the S-nitrosylation of JAK2 in both cell lines.

This result suggested that S-nitrosylation may be a regulatory mechanism for JAK2 activity.

### 2.4. Effect of GTN on IL-6-Mediated Wound Healing in TNBC Cells

**Figure 4:**
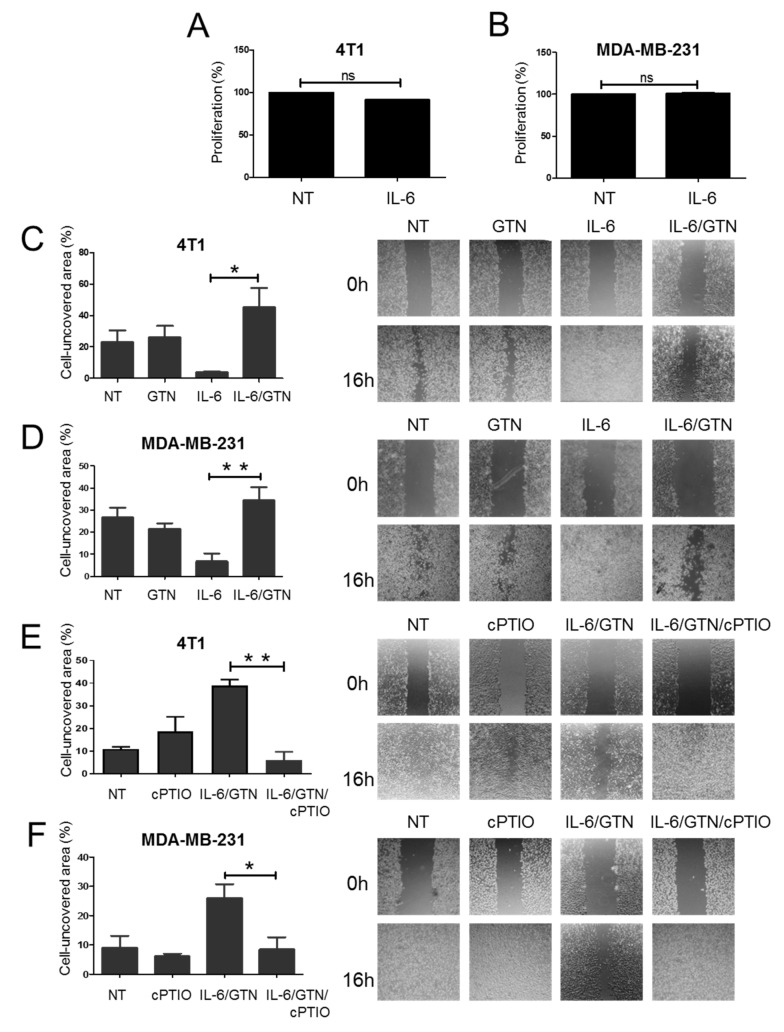
GTN prevents recombinant IL-6-induced wound healing in TNBC cells. (**A**) 4T1 and (**B**) MDA-MB-231 cells were treated with murine or human recombinant IL-6 (10 ng/mL), respectively, for 24 h and MTS proliferation assay analysis was performed. Results indicate means +/− SD of three independent experiments. (**C**–**F**) The in vitro wound healing assays. (**C**) 4T1 and (**D**) MDA-MB-231 cells were treated for 16 h with GTN (250 µM), and/or recombinant IL-6 (10 ng/mL) or left untreated (NT). Data are shown as percentage of cell-covered area. (**E**) 4T1 and (**F**) MDA-MB-231 cells were treated for 16 h with IL-6/GTN, and/or cPTIO (50 µM) or left untreated (NT). Results are expressed as mean +/− SEM of three independent experiments. Images are representative of three independent experiments. Statistically significant differences were determined using a one-way ANOVA test. * *p <* 0.05; ** *p <* 0.01; ns: not significant.

IL-6 plays an important role as a migration factor in vitro and in vivo. Wound healing assays were performed with recombinant IL-6 added to TNBC cells. To completely preclude any stimulation of cell proliferation by IL-6, we performed an MTS assay. We demonstrated that IL-6, upon 24 h of treatment, induced no modification in the rate of proliferation of TNBC cells (Figure 4A,B). We found that recombinant IL-6 increases the wound healing ability of both 4T1 and MDA-MB-231 cells (Figure 4C,D). We explored the effect of GTN on the ability of IL-6 to stimulate wound healing in TNBC cells. The wound healing activity of TNBC cells remains unchanged following treatment with GTN alone in comparison with non-treated (NT) control cells. However, combined treatment of IL-6 with GTN inhibits the wound healing activity of both 4T1 and MDA-MB-231 cells (Figure 4C,D). To confirm the role of NO in the inhibition of the wound healing by the NO donor GTN, cells stimulated with IL-6 along with GTN were treated in presence or absence of the NO scavenger cPTIO. We showed that in presence of cPTIO, the inhibitory effect of GTN on IL-6-mediated wound healing is abrogated in both 4T1 and MDA-MB-231 cells (Figure 4E,F).

Altogether, these data suggested that GTN can counteract the IL-6 responsiveness of TNBC for wound healing migration in a NO-dependent manner.

### 2.5. In Vitro Effects of GTN on Migration and Invasion of TNBC Cells

**Figure 5:**
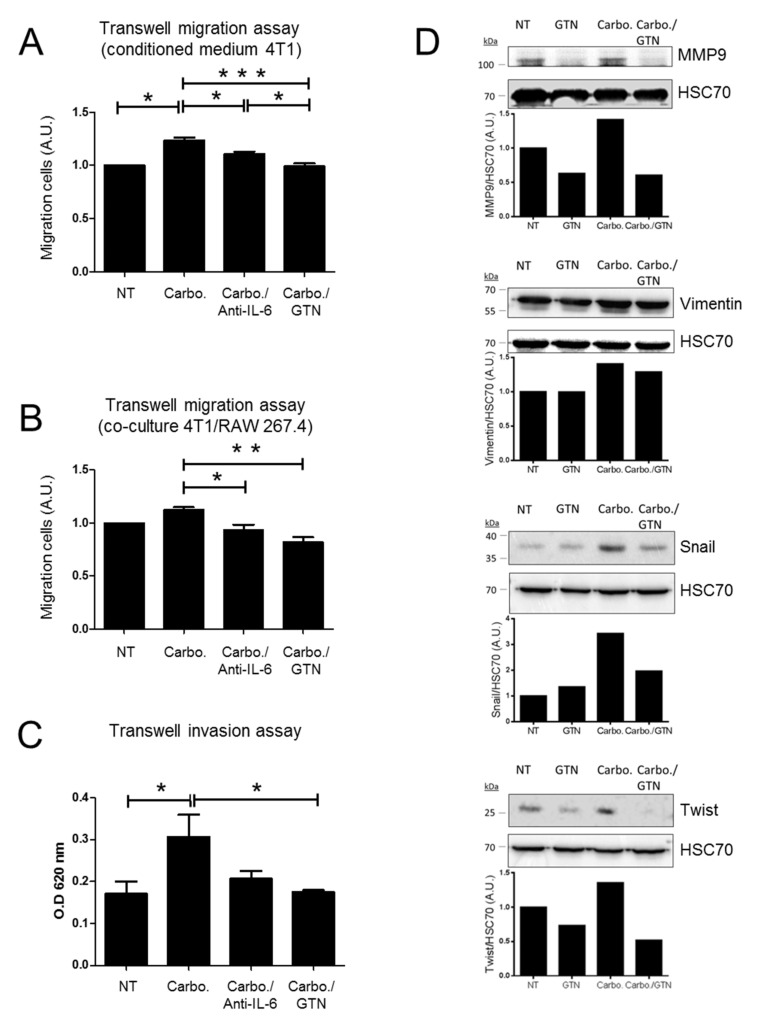
GTN prevents carboplatin-induced 4T1 cell migration and invasion in transwell systems. (**A**) Transwell migration assays were performed using 4T1 cells (upper chamber) incubated with conditioned media from 4T1 cells previously treated or not with carboplatin (10 µM) for 48 h (lower chamber). IL-6 neutralizing antibody (100 ng/mL) or GTN (250 µM) were added for an additional 16 h of treatment. (**B**) Transwell migration assays were performed using 4T1 cells (upper chamber) incubated with 4T1/RAW 267.4 co-culture (ratio 3:1) (lower chamber) treated with carboplatin (10 µM) alone or with either IL-6 neutralizing antibody (100 ng/mL) or GTN (250 µM) for 16 h of treatment. (**A**,**B**) The migration of control 4T1 cells is set at 1 arbitrary unit (A.U.) and the cancer cell migration following the indicated treatment is compared to control cells. (**C**) Transwell invasion assays were carried out using 4T1 cells (upper chamber) incubated with conditioned media from 4T1 cells previously treated or not with carboplatin (10 µM) for 48 h (lower chamber). GTN (250 µM) or IL-6 neutralizing antibody (100 ng/mL) was added within the conditioned media (lower chamber) for 16 h. Migrating and invading cells were stained with crystal violet and quantified by measuring the optical density (OD) at 620 nm. Results are means +/− SEM of three independent experiments. (**D**) Western blot analysis of MMP9, Snail, Vimentin and Twist in 4T1 cells treated with TGF-β1 (10 ng/mL) for 24 h, and then treated with GTN (250 µM) carboplatin (10 µM), carboplatin/GTN for 24 h. Densitometric analysis of proteins relative to HSC70 levels (one experiment representative of three independent experiments). Statistically significant differences were determined using a one-way ANOVA test. * *p <* 0.05; ** *p <* 0.01; *** *p* < 0.0001.

To further address cell migration, we explored the impact of GTN on TNBC cells using the transwell cell migration assay. The 4T1 cells were seeded in the upper compartment and conditioned medium from carboplatin-treated 4T1 cells was incubated in the lower compartment. To figure out whether IL-6 secreted from carboplatin-treated cells could promote 4T1 cell migration, an IL-6 neutralizing antibody was added to the conditioned medium in the lower compartment. We found significant effect of the IL-6 neutralizing antibody on carboplatin-mediated 4T1 migration activity. Moreover, the addition of GTN into the conditioned medium significantly abrogated carboplatin-mediated 4T1 migration activity (Figure 5A). We then performed a co-culture experiment using the transwell migration system. The 4T1 cells were seeded in the upper compartment while co-cultured 4T1 and RAW 264.7 cells (ratio 3:1) were seeded in the lower compartment and stimulated with the indicated treatment. The percentage of 4T1 cells migrating is more important in the presence of co-cultured 4T1/RAW 264.7 cells compared to 4T1 cells alone (data not shown). Carboplatin treatment slightly increases 4T1 cell migration. The addition of an IL-6 neutralizing antibody along with carboplatin treatment significantly affected 4T1 cell migration, and even more following GTN treatment (Figure 5B). The transwell cell invasion assays were performed with conditioned media from carboplatin-treated 4T1 cells, then treated without or with an IL-6-neutralizing antibody (lower chamber) or GTN (upper-chamber). We observed a significant increase in 4T1 cell invasion in the presence of conditioned media from carboplatin-treated 4T1 cells. Cell invasion in response to GTN was significantly reduced. In the presence of the IL-6-neutralizing antibody, there seemed to be a trend that approximated but did not reach statistical significance (Figure 5C). We investigated the role of GTN on the EMT process. The EMT was stimulated by TGF-β1 in 4T1 cells attested by the upregulation of mesenchymal markers [27]. We found that TGF-β1-induced EMT in 4T1 cells increased the level of expression of the mesenchymal markers MMP9, Vimentin, Snail and Twist, and to a further extent upon carboplatin exposure. However, in response to GTN, the level of the mesenchymal markers was reduced (Figure 5D).

Altogether, these results indicate that GTN may have a broader impact on 4T1 cell invasion than the IL-6-neutralizing antibody.

### 2.6. In Vivo Effect of GTN in a Syngeneic Model of Murine Metastatic TNBC

**Figure 6:**
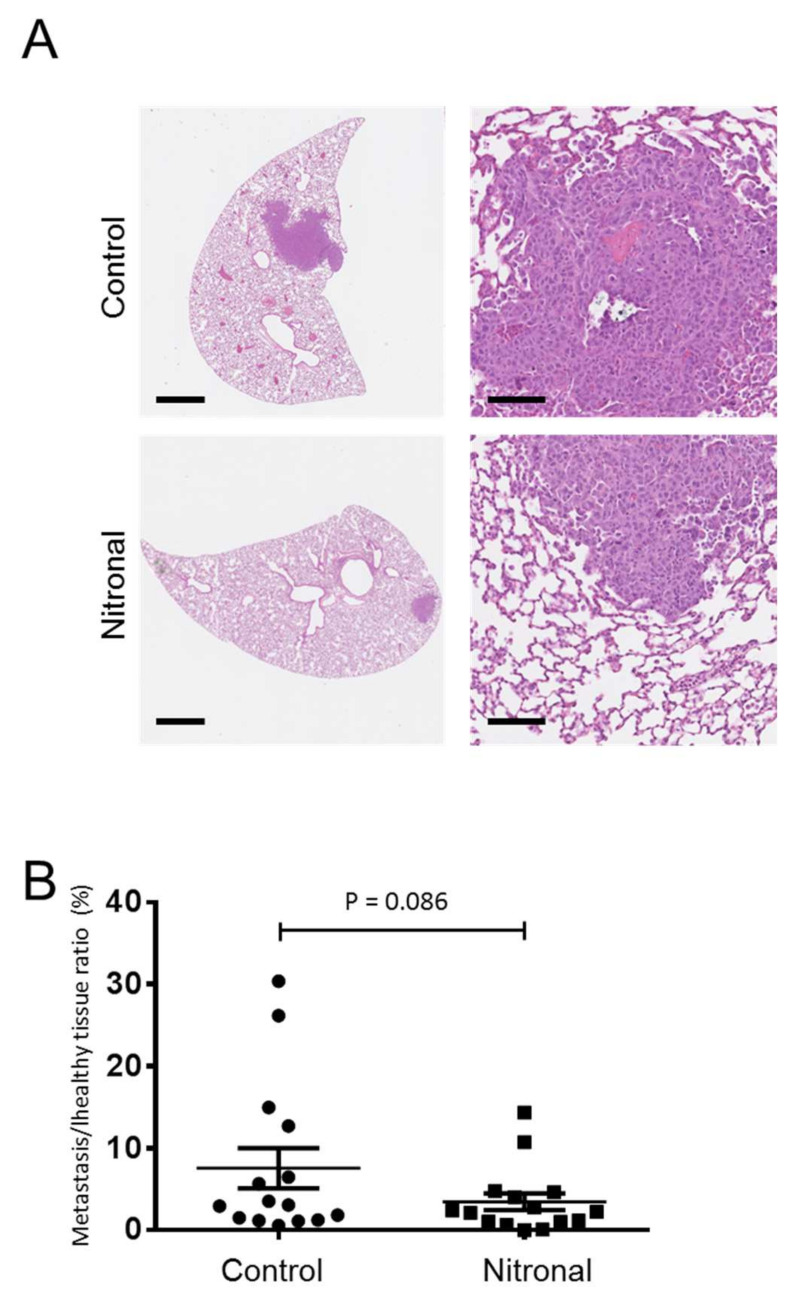
In vivo effect of Nitronal on lung metastases of 4T1 TNBC cells. (**A**) Representative immunohistochemistry images of lung sections from control or treated mice bearing 4T1 tumors. Lung metastases are visualized by hematoxylin-eosin staining of formalin-fixed paraffin-embedded sections. (**B**) Metastatic areas per lung sections for each animal were quantified as described in material and methods. Graph shows mean +/− SEM of 15 mice per group. Scale bar (left panel, 1 mm; right panel, 100 µm). Statistically significant differences were determined using a t-test with 90% confidence level and *, *p* < 0.1, considered statistically significant.

The anti-metastatic effect of GTN was undertaken in vivo using a syngeneic model of murine TNBC with 4T1 cells which metastasize to the lungs. Mice were injected with 4T1 cells, and after seven days, mice were treated twice a week with Nitronal (glyceryl trinitrate) infusion solution or saline (NaCl). After a month, we analyzed the lung tissue for the presence of metastases by hematoxylin-eosin staining of formalin-fixed paraffin-embedded lung sections from five untreated and five Nitronal-treated mice (Figure 6A). The areas of tumor lesions (% of lung sections) were evaluated for each mouse in both the control and treated groups. Compared to control mice, Nitronal-treated mice showed a decrease in the area of tumor lesions (%). The one-sided *p*-value from the Mann–Whitney test is 0.086 (statistical significance considering a 90% confidence interval (*p* < 0.1)) (Figure 6B).

## 3. Discussion

TNBC is an aggressive disease with a high rate of metastasis, poor prognosis and few therapeutic options. One current therapeutic strategy for TNBC targets DNA repair complexes (by means of platinum derivatives and taxanes). Cisplatin- or carboplatin-containing regimens (cisplatin/gemcitabine, carboplatin/paclitaxel, carboplatin/gemcitabine) are recommended for first-line and second-line therapy [28]. A platinum-based regimen including carboplatin is recommended for patients with TNBC to increase pathologic complete response [29]. However, chemoresistance to platinum derivatives arose in various types of solid cancers. In this study we show that carboplatin enhances IL-6 expression and secretion by TNBC cells to activate the JAK2/STAT3 signaling pathway and cell migration. In TNBCs and many other human cancers, IL-6 is recognized as a major cytokine produced by multiple cell types within the tumor microenvironment, and which has important roles in tumor development and progression. IL-6 is involved in various oncogenic activities (cell proliferation, cell survival, migration, invasiveness and resistance towards anticancer therapy), therefore making IL-6 signaling cascades in breast cancer a target of choice.

Therapeutic strategies of IL-6 signaling blockade have been developed for several inflammatory diseases [21,30]. However, up to now, IL-6-based therapies have demonstrated no clinical efficacy in breast cancers and also in other various types of cancer patients [30,31]. More recently, a phase II clinical trial evaluating a selective JAK1/2 inhibitor in patients with metastatic triple-negative breast cancer (tumors demonstrating p-STAT3 expression) also demonstrated limited clinical efficacy [32]. A better understanding of IL-6/IL-6 R regulation and signaling needs to be taken into account to improve IL-6-based therapies in breast cancers.

NO-releasing drugs have gained growing interest as anti-cancer agents offering new treatment options for targeting various signaling pathways simultaneously [22,33]. NO is a free radical and highly reactive molecule that exerts a dual role in cancer, either pro-tumoral or anti-tumoral [22]. Protein post-translation modification by S-nitrosylation display decisive roles in regulating several signaling pathways involved in growth, cell survival or invasion. In this study we report that GTN inhibits TNBC cell migration and invasion in vitro, most likely via the IL-6/JAK2/STAT3 axis. We demonstrate that GTN prevents carboplatin-induced IL-6 to activate the JAK2-STAT3 pathway. We also report for the first time that GTN promoted the S-nitrosylation of JAK2 which may be associated with the inhibition of phosphorylation and activation of JAK2 and STAT3. The S-nitrosylation of JAK2 had never been reported so far; however, JAK2 nitration (Tyr1007 and Tyr1008) was described to prevent its activity [34]. Strikingly, data from the biotin switch assay demonstrates that the level of S-nitrosylated JAK2 is more important in TNBC cells treated with GTN along with carboplatin, than with GTN alone. The S-nitrosylation of JAK2 most likely directly regulate its kinase activity. Our finding is in agreement with a previous report that provided evidence for a cysteine-based redox-sensitive switch that regulates JAK2 catalytic activity [35]. Furthermore, it has been demonstrated that another NO donor, S-nitrosoglutathione (GSNO), selectively S-nitrosylates STAT3 at Cys259 and inhibits its accessibility to JAK2 [36,37]. Further studies would determine whether STAT3 would be targeted in this context. Altogether, these results suggest that NO can lead to IL-6 signaling blockade. Although p53 is a downstream target of STAT3, known to be attenuated by IL-6 via the JAK/STAT3 pathway in various cell types such as LNCap (p53 wild-type) and 22Rv1 (one wild-type copy of p53 and one mutated copy of p53) prostate cancer cells [38], we did not find any changes in p53 level upon IL-6 exposure on MDA-MB-231 cells (data not shown).

Conversely, high expression of iNOS in TNBC correlates with tumor aggressiveness (progression and metastasis), chemoresistance and poor outcome [39]. The role of iNOS inhibitors on TNBC aggressiveness has been studied. Importantly, preclinical studies using the pan-NOS inhibitor L-NMMA has demonstrated efficacy in decreasing tumorigenicity of TNBC, reducing tumor growth and lung metastasis [40]. Furthermore, the use of L-NMMA in combination with docetaxel has demonstrated enhanced chemotherapy response in TNBC PDX models and represents an interesting therapeutic approach [41].

Nevertheless, the dual nature of NO in cancer, pro- or anti-tumorigenic, is still a subject of intense debate. NO action and cellular outcomes largely rely on the role of many factors in the tumor microenvironment, particularly for TNBC, a subtype of breast cancer characterized by a profound intratumoral heterogeneity.

A growing number of studies provide evidence that coordinated cytokine expression is critical for the growth and malignancy of TNBCs.

Together, IL-6 and CCL5 are key enhancers of TNBC tumor growth and metastasis [42]. Indeed, within the tumor microenvironment, IL-6 secreted by TNBC cells upregulates CCL5 expression in lymphatic endothelial cells by activating the IL-6 receptor, STAT3, which subsequently enhances transcription of the *CCL5* gene. Importantly, the simultaneous blockade of CCR5 and IL-6 receptor signaling firmly inhibits TNBC tumor growth and prevents TNBC metastasis [42].

In TNBC, the tandem expression of IL-6 and IL-8 is also critical for growth and resistance to apoptosis [43]. Importantly, several reports suggest that both IL-6 and IL-8 play a critical role in the aggressiveness of breast cancer and of other types of cancer [44]. Recently, a study demonstrated that a synergistic IL-6 and IL-8 signaling pathway is required to influence cancer cell motility and metastases. Mechanistically, the pathway likely occurs through the downstream signaling of WASF3 and Arp2/3 and subsequent regulation of actin cytoskeleton dynamics [45]. Simultaneous inhibition of IL-6 and IL-8 receptors decreases the expression of WASF3 and Arp2/3, and reduces metastasis in mice [45]. In accordance with this finding, previous clinical studies had shown that the elevated serum concentrations of IL-6 and IL-8 of breast cancer patients correlate with the stage of the disease, the recurrence, and are indicators of poor prognosis [46,47,48]. However, the prognostic role of IL-6 and IL-8 association in breast cancer may differ according to breast cancer subtype. Indeed, high levels of IL-6 and IL-8 are associated with breast cancer recurrence only among patients with HER2 tumors [12].

In our current study we found that carboplatin enhances the expression of IL-8 at the mRNA level (data not shown). However, further studies are needed to determine whether GTN could block both IL-6 and IL-8 signaling to prevent cell migration and invasion. In support to this, our in vivo studies indicate a decrease in metastatic lesions upon the NO-donor GTN. Various clinical trials to evaluate the effectiveness of GTN in a combined regimen (chemotherapy and/or radiotherapy) has been conducted [23,24,26,49,50,51]. Although some clinical benefit were observed for non-small cell lung cancer (NSCLC) patients (GTN combined with vinorelbine and cisplatin) and prostate cancer patients (GTN after surgery or radiotherapy) in phase II clinical trials [23,24], additional data are required to define how GTN should be utilized in cancer therapy. Although we cannot extrapolate to humans the concentration of GTN used for our in vitro and in vivo findings, we provide here a new rationale to overcome chemotherapy-induced IL-6 production that should be considered for future GTN-based clinical trials.

In conclusion, we demonstrated that GTN can act upstream of STAT3 activation within the JAK2-STAT3 signaling. Our study provides evidence to consider the potential efficacy of IL-6 blockade in TNBC by the NO-donor GTN.

## 4. Materials and Methods

### 4.1. Cancer Cell Lines

Human MDA-MB-231 and murine 4T1 TNBC cell lines and murine macrophage-like cell line RAW 264.7 were obtained from ATCC. All cell lines were cultured in RPMI 1640 supplemented with 10% of fetal bovine serum (FBS) (37 °C, 5% CO_2_), and were tested and certified as mycoplasma-free before use.

### 4.2. Drug, Reagents and Recombinant Proteins

The NO-releasing drug glyceryl trinitrate (GTN) was purchased from Merk (Lyon, France). Nitronal (GTN) infusion solution, 1 mg/mL (Pohl-Boskamp, Hohenlockstedt, Germany) was used for in vivo experiments. S-nitrosocysteine (SNOC), was prepared as described previously [52]. Carboplatin was obtained from the Centre Georges François Leclerc (CGFL) pharmacy. S-methylmethane thiosulfonate (MMTS), and carboxyPTIO (cPTIO) were purchased from Sigma-Aldrich and biotin-HPDP from Perbio Science (Brebières, France). Recombinant murine and human IL-6 were purchased from Peprotech (Neuilly sur Seine, France) and TGF-β1 from R&D systems (Bio-Techne, Lille, France).

### 4.3. Quantitative Real-Time Polymerase Chain Reaction (RT-qPCR)

Total RNA was extracted from cells using Trizol (Invitrogen, Thermo Fischer Scientific, Illkirch-Graffenstaden, France). Using M-MLV reverse transcriptase, random primers and RNAse inhibitor, 500 ng of RNA were reverse transcribed into cDNA according manufacturer’s protocol (Promega, Charbonnières-les-Bains, France). cDNA was quantified by real-time PCR with Sybr Green PCR Master mix (Invitrogen) on a 7500 fast Real-Time PCR System (Life technologies, Thermo Fischer Scientific, Illkirch-Graffenstaden, France). mRNA abundance was calculated according to the change-in-cycling-threshold (ΔCt) method and data represented relatively to reference gene mRNA (β-actin). Primers: hIL-6 for: ACCCCCAGGAGAAGATTCCA; hIL-6 rev: GGGTCAGGGGTGGTTATTGC; hActβ for: AGAGCTACGAGCTGCCTGAC; hActβ rev: AGCACTGTGTTGGCGTACAG; mIL-6 for: AGCCAGAGTCCTTCAGAGAGAT; mIL-6 rev: GAGAGCATTGGAAATTGGGGT; mActβ for: TTCTTTGCAGCTCCTTCGTT; mActβ rev: ATGGAGGGGAATACAGCCCC.

### 4.4. Griess Assay

The Griess assay quantifies the conversion of NO to nitrite (NO_2_^−^). Cells were seeded in 96-well plates in complete RPMI medium (200 µL/well). After 24 h and 48 h of treatment, NO_2_^−^ level was quantified by incubating 100 µL of culture supernatant with 100 µL of Griess’ reactives (solutions A and B at 1:1 ratio). Solution A composition: N-1-(Napthyl)ethylenediamine hypochlorite. Solution B composition: p-aminobenzene-sulfonamide/H_3_ PO_4_ 5%. After 15 min incubation, absorbance was read at 550 nm (corrected at 690 nm).

### 4.5. Proliferation Assay (MTS)

Cells were seeded in 96-well plates in complete RPMI medium (200 μL/well). After 48 h of treatment, cell proliferation was evaluated by adding MTS ((3-(4,5-dimethyl-2-yl)-5-(3-carboxymethoxythenyl)-2-(4-sulfophenyl)-2H-tetrazolium) (Promega). Absorbance was read after 2 h at 490 nm.

### 4.6. Apoptosis Quantification

Cells were seeded in 6-well plates in complete RPMI medium (2 mL/well). After 24 h and 48 h of treatment, apoptosis was evaluated by flow cytometry using AnnexinV-FITC/7-AAD staining (BD Biosciences, Le Pont de Claix, France). The percentage of apoptotic cells were evaluated.

### 4.7. Wound Healing Assay

MDA-MB-231 and 4T1 cells were seeded into 6-well tissue culture plate at a density that, after growth overnight, allowed cells to reach approximately 80% confluence as a monolayer. A straight scratch was made on the monolayer using a 200 µL pipette tip. Then, the cells were washed gently with PBS three times and further cultured with RPMI complete medium. Representative pictures were taken, to measure gap width at 0 h and after 16 h of incubation. Four pictures per experimental condition and time point were used to measure gap width using ImageJ software (Rockville, MD, USA)

### 4.8. Transwell Migration and Invasion Assays

Migration and invasion assays were performed using transwell systems (24-well inserts without or with Matrigel, respectively, 8 µm pore size; Corning). Briefly, 4T1 cells were seeded onto the top chamber in RPM1 complete medium. The bottom chamber was filled with RPMI complete medium (10% FBS) in the presence or absence of the indicated treatment. After 16 h, cells were removed gently from the top side of the transwell using a cotton-tipped swab. Each insert was fixed with methanol, stained with crystal violet and solubilized with 37% acetic acid. Then, optical density at 620 nm was measured.

### 4.9. Biotin Switch Assay (BSA)

The BSA was performed as described previously [53]. Briefly, 50 × 10^6^ of cells were treated with GTN or SNOC at the indicated concentration and for the indicated time. Cells were harvested, washed three times in phosphate-buffered saline (PBS) and lysed in non denaturating nitrosothiol, SNO lysis buffer (50 mM Tris-HCl, pH 7.4, 300 mM NaCl, 5 mM ethylenediaminetetraacetic (EDTA), 0.1 mM neocuproine, 1% Triton X-100) supplemented with 1 X protease inhibitors cocktail. All protein samples were normalized to 0.5 mg/mL in 10 mL of lysis buffer and free thiols were blocked by four volumes of blocking buffer (HENS buffer: 250 mM Hepes, pH 7.7, 1 mM EDTA, 0.1 mM neocuproine, 1% sodium dodecyl sulfate; containing 20 mM methyl methanethiosulfonate (MMTS)) at 50 °C for 20 min. After removing excess MMTS and washing proteins extracts twice by acetone precipitation, protein pellets were re-suspended in 1 mL of HENS buffer containing 1 mM ascorbate and 1 mM biotin-HPDP. Proteins were then recovered by two precipitations with acetone. Total biotinylated proteins were purified by precipitation on neutravidin beads and eluted with 2X blue Laemmli buffer (10 min, 95 °C) for further analysis by Western blotting.

### 4.10. Animal and Ethics Statement

Seven-week-old female Balb/c mice (total number of animals used *n* = 15) were purchased from the animal care facility at the University of Burgundy (France). The mice were acclimatized to the conventional housing system for 7 days before the period of study. All animals were observed by the animal care staff on a daily basis. Behavioral observations during the experiment were monitored by the research staff. Mice were housed at 22 +/− 2 °C in a 12 h light/dark cycle. For ethical reasons, when the volume of the primary tumor reached ~1500 mm^3^, mice are sacrificed. During the period of this study, no mice were excluded. The experiments were approved by the local Ethics Committee “Comité d’éthique de l’expérimentation animale Grand Campus Dijon” (C2 EA Grand Campus Dijon no 105) protocol code 23450.

### 4.11. In Vivo Syngeneic Mouse Model of Breast Metastasis Formation

Mice (one cage of 10 mice in a first experiment and one cage of 20 mice in a second experiment) were subcutaneously inoculated in the right flank with mycoplasma-free 4T1 cells (1 × 10^4^ cells in 100 µL) under anesthesia by continuous inhalation of 4% isoflurane gas. After 1 week, mice were randomly assigned to two groups (*n* = 5 per cage in the first experiment and *n* = 10 in the second experiment), control group (0.9% NaCl) or treated group (Nitronal^®^ 1 mg/mL, Pohl Boskamp, Hohenlockstedt, Germany). No sample size calculation was done. S.G., O.B., C.R. and S.P. were aware of the group allocation at the different stages of the experiment. Both groups were injected subcutaneously twice a week with (100 µL of either Nitronal or 0.9% NaCl). All mice received 9 injections in total (either Nitronal or 0.9% NaCl), over a time period of 29 days. After, all the mice were sacrificed and lung tissues were excised, inflated and fixed in formalin for histology analysis [54].

### 4.12. Histology and Metastases Assessment

Several sections of lungs were paraffin-embedded and stained by hematoxylin and eosin (H&E) (Histology facility CellimaP, University of Burgundy). Metastatic areas per total lung section areas were quantified using QuPath software (Version: 0.2.3, University of Edinburgh [55]). The result is reported as the percentage (%) of metastatic area to total lung area per lung section.

### 4.13. Western Blotting

Whole-cell lysates were prepared by lysing the cells in lysis buffer (150 mM NaCl, 150 mM Tris HCl, pH 7.4, 1% SDS and 1 mM sodium orthovanadate, supplemented with 1 X protease inhibitor cocktail). Viscosity of protein extract was reduced by sonication or reduced by several passages through a 26-gauge needle. Protein concentrations were measured using the Bio-Rad DC protein assay kit (Marnes-la-Coquette, France). Thirty or fifty micrograms of protein were incubated in loading buffer (125 mM Tris-HCl, pH 6.8, 10% β-mercaptoethanol, 4.6% SDS, 20% glycerol and 0.003% bromophenol blue) for 5 min at 95 °C. Proteins were separated by sodium dodecyl sulfate–polyacrylamide gel electrophoresis (SDS-PAGE) and transferred to nitrocellulose membrane (Bio-Rad). Membranes were blocked in Tris-buffered saline (TBS) containing 8% BSA at room temperature for one hour, and incubated with primary antibody diluted in TBS 0.1% Tween-20 with 2% BSA overnight at 4 °C. Membranes were washed three times in TBS 0.1% Tween-20 and further incubated with corresponding secondary antibody for one hour at room temperature. After 3 washes in TBS 0.1% Tween-20, proteins were revealed using the enhanced chemiluminescence detection kit and ChemiDoc imager (Bio-Rad).

### 4.14. Antibodies

The antibodies used in this study included the following: anti-IL-6 neutralizing antibody (R&D systems), rabbit anti-JAK2, rabbit anti-phospho (Tyr1007-10008)-JAK2, rabbit anti-STAT3, rabbit anti-phospho (Tyr705)-STAT3, rabbit anti-Vimentin, rabbit anti-Snail (Cell Signaling); mouse anti-Twist, mouse anti-MMP9, goat anti-β-actin, mouse anti-HSC70 (Santa Cruz); peroxidase affinipure anti-mouse IgG and peroxidase affinipure anti-rabbit IgG (Jackson ImmunoResearch).

### 4.15. Statistical Analysis

Statistical analyses were performed using GraphPad Prism software (version 6.01, www.graphpad.com, 21 September 2012). Significant differences were evaluated using the one-way ANOVA test or the one-tailed Mann–Whitney test used for comparing data (means +/− SEM) from in vitro experiments. For in vivo analysis, the one-tailed Mann–Whitney non-parametrical test was used to compare the control group versus the treated group (means with SEM) with 90% confidence level. *p <* 0.1 was considered statistically significant.

## Supplementary Materials

The following are available online at https://www.mdpi.com/article/10.3390/ijms22168449/s1.
