# Supplementary material for: Nitric Oxide-Releasing Drug Glyceryl Trinitrate Targets JAK2/STAT3 Signaling, Migration and Invasion of Triple-Negative Breast Cancer Cells
Source: Int J Mol Sci. 2021 Aug 6;22(16):8449. doi: 10.3390/ijms22168449 (PMC8395103; doi:10.3390/ijms22168449)
Supplement: Supplementary file 1 [file ijms-22-08449-s001.zip › ijms-1321057-supplementary.pdf]

Supplementary Figure S1

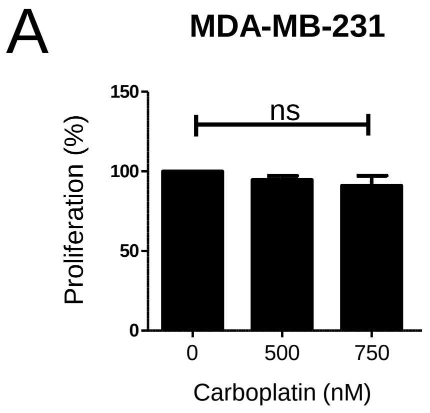

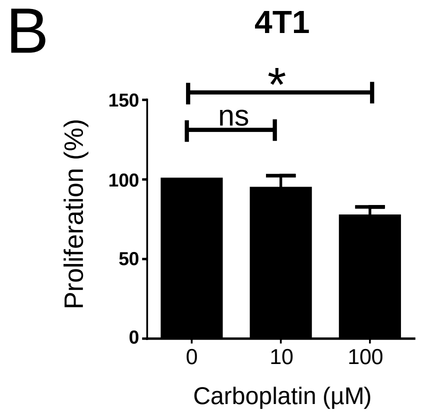

Figure S1. Effect of carboplatin on MDA-MB-231 and 4T1 cell proliferation. (**A**) MDA-MB-231 and (**B**) 4T1 cells were treated with the indicated concentration of carboplatin for 48 h and MTS proliferation assay analysis was performed. Results indicate means +/- SD of three independent experiments. Statistically significant differences were determined using a one-way ANOVA test. * $p < 0.05$; ns: not significant.

Supplementary Figure S2

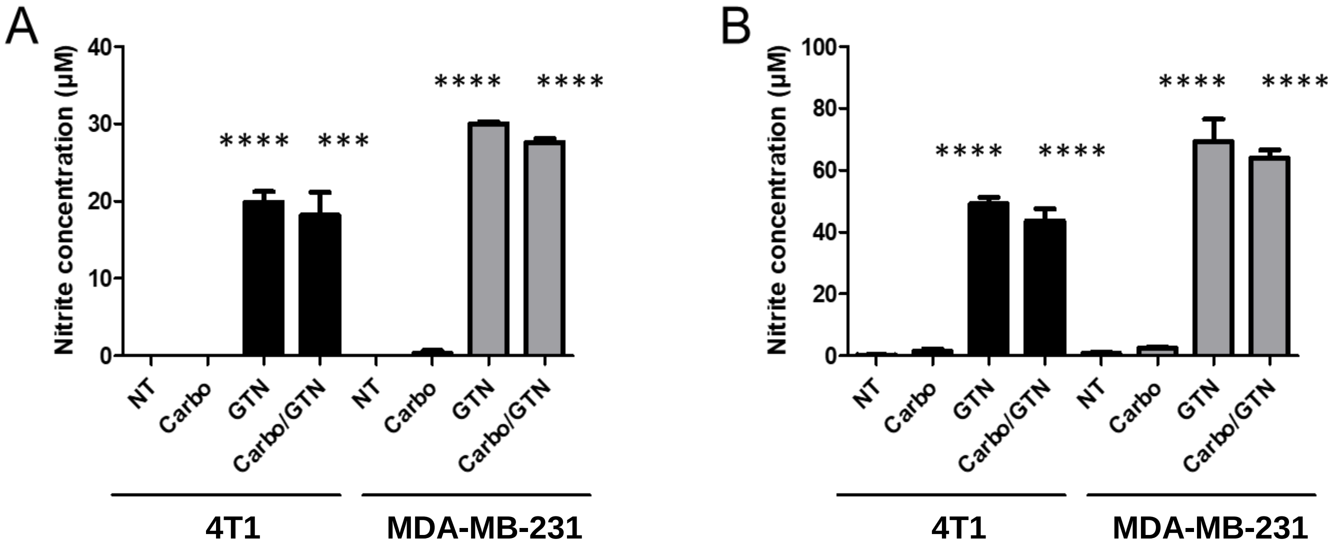

Figure S2. Determination of NO_2^- concentration. The 4T1 and MDA-MB-231 cells were or were not treated with carboplatin (10 µM and 750 nM, respectively), GTN (250 µM), Carboplatin/GTN for (**A**) 24 h and (**B**) 48 h, and the Griess reaction assay was performed to measure the concentration of the NO stable end product NO_2^-. Error bars represent SEM of three independent experiments. Significant difference from control was determined using a one-way ANOVA test followed by a Bonferroni's multiple comparison post-test. *** $p < 0.01$; **** $p < 0.001$.

Supplementary Figure S3

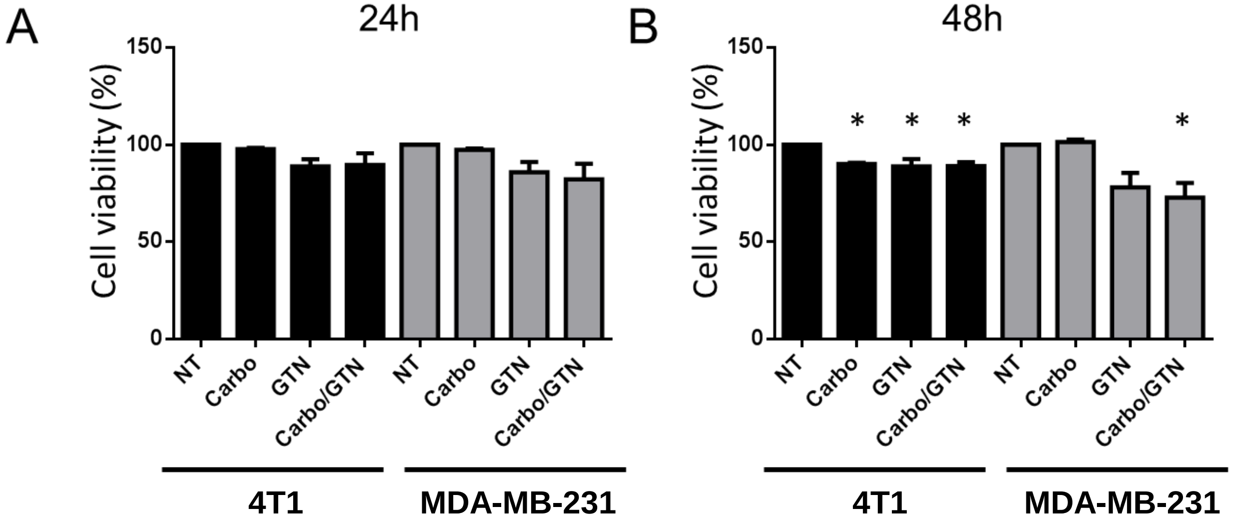

Figure S3. In vitro cytotoxicity on TNBC cell lines after treatment with GTN (250 μM) alone or in combination with carboplatin. MTS assays evaluating cytotoxicity on 4T1 and MDA-MB-231 cells treated with carboplatin, GTN or carboplatin/GTN after (**A**) 24 h and (**B**) 48 h. Experiments were performed in triplicate and repeated three times. Error bars represent SEM. Significant difference from control was determined using a one-way ANOVA test followed by a Bonferroni's multiple comparison post-test. * $p < 0.05$.

Supplementary Figure S4

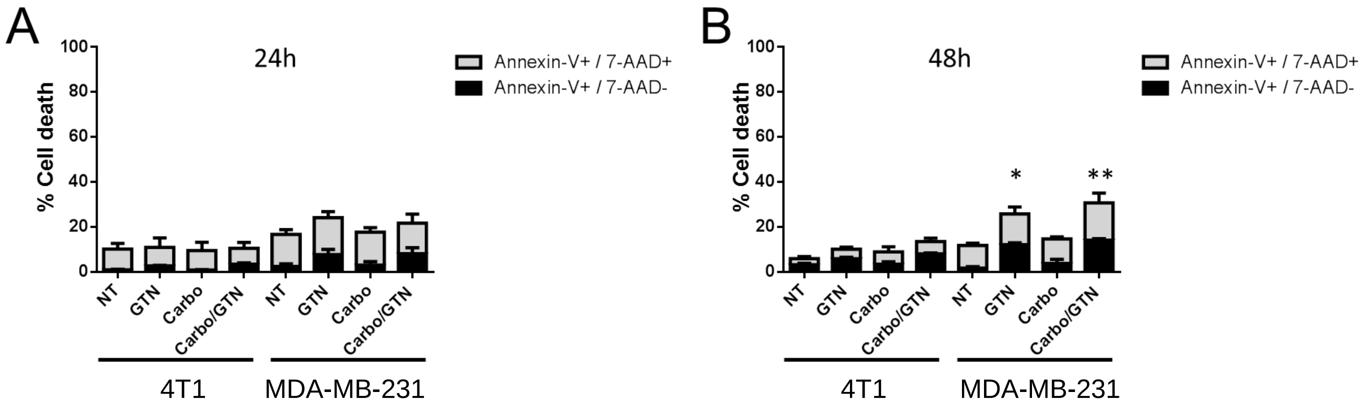

Figure S4. Effect of GTN on apoptosis in 4T1 and MDA-MB-231 cells. The 4T1 and MDA-MB-231 cells were treated with GTN (250 µM), carboplatin (10 µM and 750 nM, respectively) and carboplatin/GTN for (**A**) 24 h and (**B**) 48 h. Annexin-V/7-AAD double staining was used to assess the quantification of early apoptotic cells (Annexin-V+/7-AAD-) and late apoptotic/necrotic cells (Annexin-V+/7-AAD+). Significant difference from control was determined using a one-way ANOVA test followed by a Bonferroni's multiple comparison post-test. * $p < 0.05$; ** $p < 0.01$.
